# Monograph II. On Softening of the Brain, Arising from Anxiety and Undue Mental Exercise, and Resulting in Impairment of Mind

**Published:** 1849-10-01

**Authors:** Forbes Winslow


MONOGRAPH II.

ON

### SOFTENING OF THE BRAIN

ARISING FROM

Anxiety & Undue Mental Exercise

AND RESULTING IN

### IMPAIRMENT OF MIND.

BY

FORBES WINSLOW, M.D.

LONDON:
JOHN CHURCHILL, PRINCES STREET, SOHO.
1849.

### ADVERTISEMENT.

In deference to the kind judgment of the members of the Medico-Chirurgical Society of Brighton, I have been induced to print this essay. It is necessary, however, to premise that it was not written for publication. Having been requested to read a paper at the Society, I thought the subject of Ramollissement of the Brain, more particularly associated with Impairment of Mind, would be novel, and give rise to an interesting and a practical discussion. The essay has been sent to press as it was read. I feel it necessary to make this statement, as an excuse for its desultory character, as well as for the superficial manner in which an important subject has been treated. I append, as my excuse for printing the essay, the following resolution of the Society before whom it was read:—

"At a meeting of the Brighton Medical Society, held at the Town Hall, Brighton, on Thursday, January 4th, 1849, Benjamin Vallance, Esq., President, in the Chair, it was moved by Dr. Mackness, of Hastings, seconded by Benjamin Vallance, Esq., President of the Society,—

> "That Dr. Forbes Winslow be requested to print his excellent paper, with such additions as may tend to further illustrate the subject, in the Psychological Journal, edited by Dr. Forbes Winslow."

*Sussex House, Hammersmith,*
*July,* 1849.

### RAMOLLISSEMENT OF THE BRAIN.

---

NINETEEN YEARS ago, I submitted to the profession, through the pages of "*The Lancet*," some observations on *Ramollissement of the Brain*. Since that period, my attention has been frequently devoted to the consideration of this important, obscure, but interesting subject. Latterly, my investigations have been principally confined, in relation to this portion of the pathology of the nervous system, to those softenings of the brain occurring in the middle stage of life, often associated with great *Impairment of Mind*, and resulting, generally, from what I term an *undue taxation of the Brain and Nervous System*. I feel the more anxious to bring this subject under the notice of the profession, from having, in the course of my experience, witnessed many incurable cases of mental disorder, occurring among medical practitioners, and clearly traceable to this cause. I think we are, individually, too little disposed to recognise the necessity of a close adherence to those physiological laws which, on reflection, we must feel cannot with impunity be violated. The day of serious reckoning may be deferred; the latent mischief may not have cast the faintest shadow on our path of life; all may appear calm and secure, and yet the enemy may be creeping stealthily on in his sure but fatal progress. We know, as experienced and practical men, how insidious are the disorders of the brain and nervous system: great irremediable changes may be taking place in the delicate structure of the nervous matter, without presenting any very obvious and appreciable indications of their existence. But, alas, how often are these manifestations of health but *ignes fatui*, luring us on with false hopes of security, even at a time when the seeds of terrible disease of the brain may be in the process of germination? I have had painful opportunities of seeing some sad cases among members of our own profession, illustrating—vividly illustrating—the previous observations. I have witnessed men apparently in the vigour of health, in the full tide of professional prosperity, suddenly sink from the possession of all their mental faculties into hopeless fatuity, the consequence of an over-exercise of the cerebral functions. I have watched these cases with great and unusual interest. This impairment of the intelligence, this sudden extinction of the powers of the understanding, this change from vigorous mental capacity, to almost hopeless imbecility and drivelling idiocy, have given little or no warnings of their approach. The loss of capacity has apparently been sudden; yet upon instituting a close and searching examination into the history of the cases, I have generally been enabled to detect faint gleams of the disease, at a distance very far remote from the positive development of symptoms usually considered indicative of organic cerebral disease. This morbid change in the structure of the brain, associated with loss of mind, is not confined to those engaged in the active exercise of professional life, neither is the affection peculiar to medical men. I have seen it among persons connected with commerce, and, in fact, among all classes *whose avocations necessarily expose them to protracted anxiety and distress of mind.* I think this form of ramollissement presents peculiar characteristics. It differs in some of its features from the softening of the brain of which we read in books, and which we have been accustomed to see exhibited in our hospitals. I also think it possible to detect, at an early period, its precursory manifestations, and that, happily too, at a time when the resources of our art may be made available in arresting its onward march. In these particulars, I think it differs from ramollissement of the brain unconnected with any well-marked lesions of the intelligence, and often combined with particular and general paralysis.

Before, however, directing the attention of the Society to the species of ramollissement more particularly connected with impairment of mind, I think it necessary to consider the subject more generally.

The pathological change known as ramollissement, or softening of organic tissues, is not confined to nervous matter. The blood-vessels, both arteries and veins, are subject to the disease. Ramollissement is often perceptible in their coats. The internal tunic in each is most commonly the seat of the alteration. It is sometimes followed by ulceration. The cartilages may become so soft as to resemble paste. The osseous structure is liable to these pathological changes, producing rickets or *mollities ossium.* The centre of circulation does not escape, and we occasionally find the heart, particularly its muscular structure, after death, in a state of extreme ramollissement. Under these circumstances, the finger passes easily through its walls. The brain, then, is not the only organic structure liable to ramollissement. In considering this subject, the following points will be discussed seriatim. I think it, however, necessary to premise, that it is not my purpose to state in detail all the points of dispute among pathologists in reference to this disease. I shall confine myself principally to the most important facts, leaving the minutiæ for subsequent discussion.

I purpose considering,

1. The pathological character of Ramollissement of the Brain;
2. Its seat;
3. Its causes;
4. The period of life at which it occurs;
5. Its physical and mental symptoms;
6. Its treatment.

I then purpose directing the attention of the Society to that form of ramollissement most generally allied to *impairment of mind*—the frequent result of over anxiety and excessive mental application.

Ramollissement of the brain is understood to be, according to Lallemande's definition, "a *liquefaction* of a part of the substance of the brain, the remainder preserving its natural consistence." He lays a stress on the word "part," because, as he observes, if the whole of the brain were soft, we should have some difficulty in deciding whether the change was pathological in its character. Softening may, as Dr. Watson observes, vary in degree, from the consistence which naturally belongs to the cerebral substance to that of thin cream. In its minor degree, it may be easily overlooked, and is more perceptible to the touch than to the eye; the cerebral matter is less coherent, but is not yet discontinuous or broken down. It may be washed away, however, by letting a slender stream of water fall upon it, and then the softened parts are easily distinguishable from those which retain their natural consistence. The *vexata quæstio* among pathologists has been, whether ramollissement of the brain be a disease *sui generis*, or the result of inflammation. This point has been the subject of much discussion, the combatants being Rostan, Lallemande, Andral, Fardel, Calmeil, Abercrombie, Bennett, &c., and other distinguished pathologists of Germany, France, and England. Without going over ground which has been so frequently traversed, or even recapitulating facts which must be familiar to all the members of this Society, it will be only necessary for me to state the results to which, after a careful examination of the question, modern pathologists have arrived. The views of Abercrombie are, I believe, those generally received by the profession. This distinguished writer generalizes the whole subject by observing, that ramollissement of the brain may occur under two modifications essentially different from each other. The cases referred to by Rostan, who considers this affection as *sui generis*, although admitting that it is sometimes the result of inflammation, occurred almost exclusively in *persons advanced in life*. It was generally associated with attacks of paralysis and apoplexy, and often found combined with extravasation of blood. In many of the cases, the softening surrounded old apoplectic cysts. The inflammatory ramollissement of which Abercrombie speaks, was seated in the more central parts of the brain, the *Fornix*, *Septum Lucidum*, or *Corpus Callosum*, being generally implicated in the organic lesion. The ramollissement was generally preceded by well-marked attacks of inflammation, and occurred in persons at an early period of life. The *post-mortem* appearances were generally deep redness surrounding the ramollissement, suppuration, and deposition of false membranes. Dr. Abercrombie, in his attempt to reconcile the views of Rostan with his own, observes that the peculiar softening of the cerebral matter is analogous to the gangrene which occurs in other parts of the body; it may be the effect either of inflammation, or be the result of a *starvation of the brain*, caused by a diminished supply of arterial blood being transmitted to that organ, in consequence of the calibre of the cerebral vessels being altered by disease. The ramollissement described by Abercrombie is said to have been caused by the former, and that referred to by Rostan, by the latter condition. We know how often ossific deposits take place in the arteries in advanced life, and therefore we need not feel surprised at ramollissement being the frequent consequence of the failure of the circulation caused by the mechanical obstruction to the free passage of the blood through the brain. Dr. Hughes Bennett (who has entered scientifically into a consideration of the subject) maintains, that by the aid of the microscope, he is able easily to recognise the inflammatory and non-inflammatory softening of the brain. The former is said to be characterized by the presence of exudation corpuscles, and granules. In the non-inflammatory ramollissement these bodies are never found. He also has discovered, as the result of his microscopical observations, that in inflammatory softening there exist the formation and development of nucleated cells in exuded blood pliasmæ. The non-inflammatory ramollissement consists, according to the same authority, "in the mechanical destruction or maceration of the nervous tissue in serum, or is the result of putrefaction." The yellow and white softening is said to be the effect of inflammatory action. The fawn-coloured ramollissement is generally consequent upon an opposite condition. The other conclusions of this able physiologist I give in his own words:—"That red softenings usually depend upon congestion or the direct extravasation of blood; yellow softenings, on the imbibition of the colouring-matter of the blood; fawn and grey-coloured softenings, on the presence of grey exudation corpuscles; and white softenings, in the great majority of cases, are *post-mortem*, and the result of maceration in serum.

"In no single instance has softening of the nervous centres been traced to the presence or infiltration of pus.

"That inflammation of the central parts of the brain generally produce well-marked lesions of sensation and motion; whilst in inflammation of the peripheral portions, lesions of intelligence are commonly well pronounced.

"That in idiopathic inflammatory softening of the brain, contraction in one or more limbs is a common symptom.

"That the fawn-coloured spots described by Dr. Sims, are no evidence of the cure of inflammatory softening.

"That inflammation accompanying hæmorrhage is usually consecutive.

"The softening surrounding apoplectic clots, or sanguineous infiltration, is no proof of inflammatory action."

Ramollissement of the brain is not confined to one portion of the cerebral mass. The parts most frequently found in a softened state are the grey matter of the convolutions, the *Thalami* and *Corpora Striata*. The

*Corpus Callosum*, *Septum Lucidum*, and *Fornix*, are also often the seat of this pathological change. Andral has published an analysis of 117 cases. The following is the result:

| | |
|---|---|
| Softness of the entire Hemisphere . . . . . . | 4 |
| ——— of only one Hemisphere in its entire extent | 13 |
| ——— of the convolutions alone, and others more deeply seated . . . . . . . . . . . . . | 14 |
| Anterior Lobes . . . . . . . . . . . . . | 27 |
| Posterior „ . . . . . . . . . . . . . | 37 |
| Corpora Striatæ . . . . . . . . . . . . . | 28 |
| Optic Thalami . . . . . . . . . . . . . | 15 |
| Parietes of the Ventricles . . . . . . . . . | 2 |
| Cerebral Peduncles . . . . . . . . . . . | 1 |
| Dispersed through various parts . . . . . . . | 5 |

This pathologist does not think, as some suppose, that the grey substance of the brain is more frequently softened than the white. I have compared this table with others published by Calmeil, Fardel, and Abercrombie, and have found but little difference in the results at which they have arrived. It will, therefore, be unnecessary for me to go further into this subject. I shall merely observe, as a fact of some pathological importance, that it is established, that those portions of the brain most liable to softening are generally the seat of cerebral hæmorrhage.

I come now to the consideration of the causes of ramollissement of the brain. These are various. In inflammatory ramollissement occurring in young persons of a plethoric constitution, the disease is generally the result of those causes which develope inflammatory action, and is often seen as the effect of physical injury to the head, and an extension of disease from the internal ear to the brain. In these cases, we witness all the symptoms which precede inflammation of the brain or its membranes. The more frequent causes, as far as they can be ascertained, of this morbid change, are, (I am now speaking without reference to age,) physical injury to the head at an early period of life, exposure to intense cold or heat, defective nourishment, syphilis, abuse of mercury, excessive venery, retrocession of acute cutaneous eruptions, debility of constitution, habits of intoxication, self-abuse, opium-eating, continued irritation of the stomach and bowels, and mechanical obstruction to the free circulation of blood through the brain, from tumours, or from osseous or fibrous matter deposited in the arteries of the brain, particularly at the base of this organ. The moral or mental causes are, anxiety of mind, and an overstraining of the functions of the brain. The diseases with which ramollissement of the brain is frequently associated, are, valvular affection of the heart, aneurismal tumours on the large vessels, rheumatism, gout, &c. Considerable softening of the brain is sometimes noticed very early in life. It may be detected during all the periods of childhood, the result of active cerebral disease. Of 153 cases of ramollissement referred to by Andral, 39 were forty years of age, 54 between forty and sixty-five, 60 between sixty-five and eighty-seven. It is mentioned as a singular fact, that the period of life when the number of the population is lowest, is, nevertheless, that which gives the highest absolute number of cases of ramollissement.

In considering the symptoms of ramollissement, I shall first speak of those which are precursory. In doing so, I purpose confining my observations to cases occurring in the middle or advanced period of life. Ramollissement taking place at an early age, is generally associated with acute cerebral affections, and the indications are those of active disease going on in the head. It is a question among practical men, whether there are any well-marked characteristic symptoms which may be considered precursory of ramollissement of the brain? It is most important to detect these, if they exist; for at this stage of the malady, it may be possible, by a well-directed application of the agents placed at our disposal, to impede the progress of the disease, and often to cure it entirely. I think it is our duty to trace, if possible, the *first warnings* or approach of this affection. I am always anxious, when consulted in these cases, to ascertain, if possible, the symptoms which have been premonitory of the cerebral disease; for in the early stages, when the disturbance is independent of any extensive organic alteration, and merely the effect of an altered action of the functions of the part, the disease admits, *cæteris paribus*, in the majority of instances, of an easy cure. If the symptoms be mistaken or overlooked, and the affection be neglected in its earlier or incipient stages, little or nothing can be done when the disease in all its formidable characteristics manifests itself. But even in cases where the disease appears to be fully developed, it is possible to effect a cure; if not, it is often in our power to mitigate considerably or alleviate its distressing symptoms.

Attacks of acute ramollissement of the brain are generally preceded by all the symptoms indicative of the consecutive progress of inflammatory affections of this organ. If ramollissement of the brain be the result of the congestion of that organ, it is most important to keep in recollection the symptoms characterizing this state of the vascular system. I believe, in many cases, the softened state of the nervous pulp is the effect of congestion, and congestion only. It is, therefore, of much consequence to detect in its earliest stage this premonitory sign. Before, however, speaking of congestion, we must revert to those symptoms which are precursory of this condition of the blood-vessels. We have first what is termed "cerebral determination:" this is well understood. Following this, there is an active cerebral congestion, consequent upon the blood not being carried off by the veins as rapidly as it is introduced by the arteries. There is, therefore, an accumulation of blood in the arterial system; but should there be a deficiency in the contractile power of the cerebral capillary vessels, interfering with the circulation, a condition denominated "passive congestion" takes place. This may also result from any mechanical impediment to the return of the venous blood from the brain. In active cerebral determination, the functions of the brain are *excited*, or *exalted*, resulting, it is supposed, from the increased arterial action without congestion. The cerebral powers are *depressed* in active, or arterial congestion, consequent upon an interruption to the free circulation of blood in the brain. In passive, or venous congestion, the cerebral functions are also in a state of *depression*. As the conditions here spoken of are, in ninety-nine cases out of a hundred, the precursory symptoms of inflammation of the brain, how important does it become that we should be on our guard whenever any of these signs are present. The symptoms of congestion of the brain are so well known, that it is unnecessary for me to recapitulate them.

I now approach the consideration of the precursory symptoms. In the majority of cases I have usually found the early signs to be headache, the pain being often circumscribed. This headache I have known to be of years' duration. Conjoined with it we have vertigo, imperfect vision, a sensation of weight in the head, increased temperature of the scalp, irregular action of the superior palpebræ muscles, double vision, optical illusions, a want of sensation in the scalp. After careful inquiry, I have generally found the symptoms just enumerated to be those which, in reference to the head, are frequently the precursors of this organic change. In some cases, no headache has been complained of. Following these symptoms, we generally have a sense of numbness accompanied with an irregular action of the organs of voluntary motion. I have noticed a diseased sensation, irregular muscular action and mere loss of power in the muscular system to precede for some period the development of the well-marked and characteristic signs of softening. Whenever head-symptoms present themselves, we should watch, from day to day, the condition of the *muscular power*. In some cases, we are able to discover the first symptom of diminished motor power long anterior to the development of absolute paralysis. *Muscular debility is generally precursory of irregular muscular action or deficiency of motor power.* The patient suffering from head-symptoms will complain of want of tone in the muscles; he will find himself incapable of taking his usual extent of exercise; will often feel under the necessity of sitting down whilst out walking. Conjoined with this debility there is a numbness of some portion of the body, which is generally attributed to an imperfect circulation of the blood in the part. Following this want of muscular tone, the patient complains of occasional weakness of the leg or ankle coming on suddenly whilst taking exercise. He will when walking lose, perhaps only for a moment, complete control over the muscles of the leg, or his ankle-joint will give way, leaving the person to suppose that there exists a weakened or diseased state of the ligaments of that joint. I have particularly noticed these symptoms in four well-marked cases of ramollissement. For some period before the medical attendant had any suspicion of the existence of serious cerebral disease, this tendency on the part of the ankle-joint to yield was specially noticed. This symptom often manifests itself for years, and may be quite independent of disease of the brain. It is only when it occurs in combination with other symptoms that it becomes of importance as a diagnostic sign. As the disease advances, other indications of a less equivocal character present themselves. The speech becomes affected. In reference to this symptom, it is interesting as well as important to regard the first warnings or scintillations of an altered action in the vocal function. Before the thick, husky tone of the voice, or the tremulous state of the muscular fibres of the tongue excite alarm in our minds, we may detect *a loss of voluntary power over the ideas* and an inability to pronounce certain letters in the alphabet, particularly the letter *R*. I believe this phenomenon always precedes the physical signs of which I have been speaking. As this may properly be considered as a mental symptom, I shall reserve what I have to say upon the point until I come to the consideration of that part of my subject.

According to the experience of men who have had ample opportunities of studying the early or incipient symptoms of ramollissement of the brain, the following are considered to be the usual symptoms of its approach, or actual existence. A sense of debility over the whole body, of heaviness, numbness, and loss of power in the extremities, usually of one side; a muddy, pallid complexion. The morbid sensations in the extremities are often of some duration, the patient having a constant sensation as if the limbs were asleep. They drag in walking, and the patient is unable to use the arm and hand with as much freedom and strength as usual. But more frequently these symptoms are only occasional, coming on in paroxysms, in which the extremities of one side suddenly fail, and the patient must either sit down or fall, but in a few seconds or minutes is again able to rise and pursue his way. In addition to these symptoms, the patient frequently suffers from headache, giddiness, stammering, dimness of sight, visual spectra, noise in the ears. The circulation and vegetative functions are undisturbed during this stage.

It has been maintained that in the early period of ramollissement there exists a permanently contracted state of the flexor muscles of one or more of the limbs. In some cases, the contraction amounts only to a slight stiffness; in others, it reaches such an extent, that if the arm be the part affected, the hand is clenched and remains pressed against the shoulder; or if the leg, the heel is carried up to the hip. I attach no importance to this as a diagnostic sign. It is often present in affections of the membranes of the brain, in encysted abscess of the brain, and it is frequently associated with typhus fever when the cerebral disturbance is great. Of the more advanced symptoms it is unnecessary for me to speak. In conjunction with the physical signs previously enumerated, we have certain mental indications the effect of these pathological changes. I am now speaking of the ordinary attacks of ramollissement. In these instances, the mind is generally more or less affected, the symptoms being, a loss of memory, of the power of attention, and change of temper. It is difficult to account for the fact of the memory being the first faculty of the mind to give way in this cerebral affection. But it is a remarkable circumstance that in ramollissement of the brain, one of the earliest mental signs is a weakened power of recalling to the mind recent impressions. Whenever the mind has been overworked, and I find the memory failing, my serious apprehensions are excited, and without creating unnecessary alarm, I invariably enjoin an immediate cessation from all mental exertion. The symptom, however, more particularly deserving of notice, is the loss of voluntary power over the ideas, and the disposition to substitute one word for another. I have so often seen this symptom precede those which are generally regarded as the pathognomonic signs of ramollissement, that I consider it my duty to make it the subject of special remark. The substitution of one word for another is a remarkable premonitory symptom. This symptom is often precursory of paralysis; *the paralysis of ideas appearing to precede that of the tongue.* A patient threatened with an attack of the disease has been known, during conversation, to misplace his words; for instance, if he wished to ask for bread, he has asked for butter, and *vice versâ*; and so with reference to other things, being angry with himself for his apparent absence of mind. A gentleman who appeared apparently in excellent health, manifested this symptom for several days, much to the annoyance of himself and those about him. It excited no uneasiness in the mind of those who were witnesses to the irregularity of thought. About a week afterwards, whilst sitting at the breakfast-table, he was suddenly seized with paralysis, of which he ultimately died. Extensive ramollissement in the central portion of the brain was detected after death. It must likewise never be forgotten that this symptom is often precursory of an attack of apoplexy.

What can I say on the subject of treatment? Except in the early stage of this formidable disease, how vain are all our efforts to impede its march? It has been affirmed that ramollissement of the brain admits of cure even after changes in the structure of that organ have taken place. Pathologists have maintained that by the aid of the microscope they have succeeded in discovering evidences of softening of the brain which must have existed some years previously to death, and which had been cured. Without questioning the authority of these writers, I must confess that I cannot from personal observation bear testimony to the accuracy of the statement. I have seen cases presenting all the features of ramollissement of the brain recover, but I have never been able to detect in *post-mortem* examinations the evidences of which pathologists have spoken. If, however, this view be correct, it encourages us to proceed in our treatment, even where the symptoms of ramollissement are obvious and well-marked. In the incipient stage of softening, it may be possible, by well-devised means, to arrest the disease. The treatment must be adapted to the peculiarity of each case. In persons of plethoric habit, with decided indications of active disease in the head, cautious depletion will be necessary. The treatment must be conducted on general principles. Unfortunately, we possess no *specifics*

for this affection. The great question in reference to this part of the subject is, whether this morbid lesion does not often commence in the *peripheral extremities of the nerves spreading towards the centre of the nervous system, producing ultimately the organic change to which I allude?* Dr. Graves, of Dublin, takes this view of the subject, arguing that it is possible for this to occur. Certainly many of the cases so minutely detailed by Rostan are examples of what is termed *Creeping Palsy*, being illustrations of disease spreading from the extremities of the nervous system towards the centre. Under such circumstances, it may be practicable to cure the disease before the nervous centres are implicated in the destructive process. This is a point worthy of our profound consideration. The points of physiological, pathological, and practical interest in relation to these attacks of local paralysis, at a distance from the great nervous centre, and independent of disease in that quarter, are, upon what state of the nerves does the loss of power depend?—is it often a muscular affection interfering with the normal action of nervous power, or an alteration in the tissue of the nerve itself, and what treatment is likely to be successful under these circumstances? I reserve, for the concluding portion of my observations, the remarks which I purpose making in reference to the mental treatment of this cerebral disease. Having spoken of ramollissement of the brain generally, I now come to the consideration of softening specially associated with mental impairment. Of the precise pathological character of this species of morbid alteration, it is extremely difficult to speak. I do not consider it to be, in many cases, the result of inflammation; as a rule, it is connected with an anæmiated condition of the system. It manifests indications which lead me to the conclusion, that we may with propriety consider it a disease *sui generis*, a something apart from those forms of ramollissement associated with profound coma, or with lesions, of the motor power. It is not confined to persons in advanced life, and often exists for years independently of paralysis of the extremities. It frequently developes itself at a period anterior to the time when we are justified in supposing the circulation of the brain to be impeded by osseous deposits in the blood-vessels. In fifteen cases which have come under my own care, in which I had every reason to believe that this condition of the brain existed, I found that the majority occurred before the age of forty, and in every instance the mental powers had been most severely taxed. In this form of ramollissement, the pathological change will generally be found situated in the cortical part of the brain. The other portions of the cerebral mass may be implicated, and often are seriously so, in the destructive disorganization, but I have never seen softening of the brain associated with well-marked lesions of intelligence unaccompanied by organic changes in that region of the brain generally admitted by physiologists to be the seat of the intellectual principle. Few physiologists are disposed to deny that the mental powers are closely connected with the cortical or cineritious substance of the brain, or, as Mr. Solly terms it, the "hemispherical ganglia." The medullary portion of the brain is merely the passive servant of the cineritious part, either as the conductor of its commands to the muscles, or of various impressions made upon the extremities of the nerves of sense, which it, the cineritious, perceives, and with which it works.

It is extremely difficult to generalize satisfactorily the peculiar symptoms of this description of morbid alteration. Each case generally presents its own individual characteristics. I may, however, observe, that the attacks were not often—until the disease became much more advanced—connected with a morbid condition of the motor power. In all the cases of softening occurring at an early period of life, I have almost invariably found the morbid condition of brain consequent upon excessive mental labour or anxiety of mind. In four cases, the parties affected had been guilty of onanism to a great extent; in three cases, the system had been frequently salivated with mercury; and in two they had been exposed for a protracted period to the intense heat of the sun without absolutely having a *coup de soleil.* Superadded to these causes, predisposing the persons to morbid cerebral action, there was an undue exercise of the functions of the brain, occasioned by either excessive application to business or study, or over-anxiety of mind, consequent upon pecuniary or other losses. In most of these cases, the physical symptoms, although they had been overlooked, were nevertheless well-marked, and had existed in several of the cases for some years before evidence of any mental affection presented itself. When the powers of the mind, or, speaking physiologically, the functions of the brain, are exposed to severe and continued exercise, we cannot be too careful in watching the head symptoms. Of the common physical signs of the approaching mischief I have previously spoken.

In three cases which came under my notice, the only symptom of any importance, which existed for four years before the disease became more fully developed, was an intermittent pain, severe in its character, in the posterior part of the head. For the removal of this pain various remedies were tried, but nothing appeared to do good. I could not ascertain that there existed any other indication until well-marked signs of organic disease of the brain were exhibited. In the majority of the instances of ramollissement associated with lesions of intelligence, the physical symptoms have been so trifling and insignificant as almost to escape observation. Occasionally they were not noticed until the mind had given way. In cases where the functions of the brain have been severely taxed, headache, feelings of uneasiness in the brain, sensations of weight in the posterior part of the head, accompanied with a disturbance of the digestive functions, a tendency to spasm, and a loss of muscular power, ought never to escape our serious consideration. These physical signs are generally the sure precursory manifestations of ramollissement of the brain. I am now speaking in general terms. As a rule, each case presents a physiognomy peculiarly its own, it being extremely difficult to fix upon any one or two symptoms characteristic of this organic lesion. In one case of severe ramollissement, the patient had never suffered at all from cephalalgia. We all know what serious mischief may be going on in the brain without giving rise to pain, or even to uneasiness in that quarter. I feel I may be somewhat premature in observing that the ramollissement of the brain allied to extensive lesions of the intelligence, or mental impairment, is an affection *sui generis* in its character. I am of opinion that the pathological change commences earlier than most writers are inclined to admit. Are there any reasons why it should not? I believe it is impossible to detect, in many cases, the presence of ramollissement of the brain without a minute microscopical examination. In most cases of cerebral organic disease, the mind cannot be otherwise than affected. I know that pathologists, who are disposed to question the close relationship which exists between the operations of the mind and a sound condition of the cerebral organs, are in the habit of citing cases in which the brain has been found extensively disorganized after death, the mind being intact during the existence of these morbid changes. It is true the intellectual and perceptive powers may not exhibit any aberration or derangement, but it is a psychological and a pathological absurdity to suppose, that any organic lesion can take place in the encephalon, involving the cortical structure, without affecting the *powers* of the mind.

What are the incipient mental symptoms of this form of softening? I can best reply to this interrogatory by referring to the history of several cases which have come under my notice. Without troubling the Society with a minute history of each, I may observe that the following have been the premonitory mental symptoms usually manifested under such circumstances. The patient has complained of a weakness of mind, depression of spirits, disposition to cry, a feeling of mental languor, of an inability to exercise continuity of thought, of a deficiency of concentrative power, and a weakened condition of the faculties of volition and attention, confusion of ideas, and defective memory. Experienced men lay great stress on this latter symptom, as being, *cæteris paribus*, the sure forerunner of this condition of brain. I was consulted, not many days back, by a person who had filled for many years the situation of commercial traveller, and had been exposed to great anxiety of mind. The principal mental symptom under which he laboured was a *total loss of memory for recent occurrences*. I have no doubt, from the history of the case, that ramollissement of the brain had commenced its ravages.

A physician who had been engaged, for a period of twenty years, in active practice had been subject for a few years prior to his attack to an unusual degree of mental excitement and hard work. His wife urged him repeatedly to retire from the active duties of his profession, but he declared his intention of dying in harness. The first symptom which he manifested was his mistaking the names of two sisters whom he attended, an unusual thing for him, as he was generally so very particular and precise in all his transactions with them. I was consulted in this case, when, alas! I could hold out no hope of recovery. His wife informed me that before the mental confusion became so apparent as to attract the patient's own observation, she had noticed an alteration in his manner, which made her fearful that he was overstraining the powers of his mind. He was observed to be more than ordinarily restless and fidgetty, forgetting his appointments, anxious about trifles, and apprehensive of being left in adverse circumstances. This alteration in his mental powers was perceptible eight months before the family would believe that anything serious was portending.

A gentleman engaged on the Stock Exchange had for some months been exposed to great perturbation of mind. His brother, who was associated with him in business, perceived an alteration in the mind of his relative, and asked my opinion of the case. The symptoms were an undue anxiety about trifles, an inability to attend, as usual, to the minutiæ of business, and defective memory. It was evident that the brain had been overworked. I recommended immediate REST. I found that conjoined with the impairment of the intellect there was considerable derangement of the general health, particularly of the hepatic functions. I urged the necessity of his retiring altogether from the anxieties of speculation for six months. This advice was followed; his general health was restored by the ordinary means. He took gentle exercise on horse-back daily, and at the expiration of seven months his mind had completely recovered its vigour. The patient complained of a sensation of fulness in the head; and as the pulse was of a character to denote activity of circulation, I had a few leeches applied to the neighbourhood of the occiput with much advantage.

A lady who had the management of a large school was placed under my care, presenting, as I thought, unequivocal symptoms of the commencement of softening of the brain. In addition to a positive impairment of the mind, rendered remarkable by an almost total loss of memory for recent occurrences, she had an amaurotic affection, accompanied with a slight affection of the motor power. I treated the case on general principles, applied counter-irritants to the head, regulated all the secretions, brought the system slightly under the influence of mercury, and gave small doses of the tincture of lyttæ. This mode of treatment, conjoined with regular exercise in the open air, mineral tonics, and the employment of moral means, effected a permanent cure.

A medical gentleman, a general practitioner, was placed under my care by Dr. Conolly. He had for some years been occupied in conducting an extensive country practice. Not satisfied with the amount of anxiety necessarily resulting from his professional labours, he was in the habit of sitting up until two or three o'clock in the morning engaged in study. His mind soon became impaired, and committing some acts of extravagance, whilst out visiting his patients, he was detained by a magistrate, and, with the consent of his family, was sent to a county asylum. In the course of a few weeks he was transferred to me. The case gave unequivocal indications of great mental debility, with obvious incipient paralysis. There could be no doubt of the nature of the case. All who saw the gentleman pronounced him to have softening of the brain. In eight months he left me perfectly restored. Six months after, he came to London for the purpose of consulting me relative to a practice, the purchase for which he was negotiating. He continued well. In this case, in addition to the affection of the mind and the loss of power over the voluntary muscles, there was paralysis of the sphincter ani. Notwithstanding all these most alarming symptoms, this gentleman was restored, re-entered his profession, and is now engaged in the exercise of its responsible duties. Alas! these cures are not of common occurrence.

A gentleman, whose property was made the subject of vexatious and protracted litigation, presented evidences of great impairment of mind. The first symptom noticed was the habit of extreme abstraction, which was most unusual in him. He would sit for twenty minutes at a time with a fixed look, staring at vacancy. His bodily health appeared unaffected. He was physically vigorous, indulged in great exercise, and was able to take an active part in athletic games. His mental peculiarity was the only symptom which alarmed his family. I saw the case with other practitioners, and he was immediately subjected to treatment; but notwithstanding the prompt and, it was hoped, efficient measures pursued, the disease gradually advanced until it was developed in all its intense and incurable malignity, and the poor man, in the prime of life, sank into loathsome and hopeless imbecility. In this case, the mind was not the subject of aberration or delusion. It was broken down by great anxiety. It is the absence of everything like *derangement* of the intellect which gives a peculiarity to the cases of which I am speaking. Occasionally the patient mistakes the wanderings of his imagination for realities; but such instances form the exception and not the rule. I am anxious to confine my observations to those cases in which the general powers of the intellect appear reduced in vigour and power, and in which insanity (in the ordinary acceptation of the term) does not exist.

A distinguished member of our own body had been engaged for many years in the anxious and responsible duties of an active professional life. His mind gave way. The first alarming indication was, the unusual degree of solicitude he manifested in reference to the accuracy of his prescriptions, frequently writing and re-writing them, repeating questions to his patients, and forgetting the names of his most intimate friends. Conjoined with these symptoms there was great irritability of temper. Before friends, however, noticed the phenomenæ I have referred to, there existed evidence of an over-worked mind, clearly indicating the necessity of great caution in the exercise of its powers.

A gentleman, aged twenty-five, who had exposed himself to intense mental application for a period of twelve months, with the view of taking honours at one of our universities, was noticed one day to manifest an extraordinary degree of risibility. He burst into a fit of laughter in the presence of a number of college friends, nothing having been said to excite anything like pleasantry or merriment. The fact was noticed by one of his most intimate associates, and caused some anxiety. He subsequently became depressed and sullen, taking little notice of anything. He was placed under treatment, and was finally consigned to an asylum. The symptoms of depression, conjoined with extreme feebleness of intellect, continued for some years, before any symptom resembling paralysis presented itself. The disease then exhibited itself in full maturity, and he became as helpless as a child. In this case we perceive the commencement of the disease at the early age of twenty-five, the result of undue taxation of the powers of the mind. It may be a question whether the softening, which subsequently manifested unequivocal signs of its presence, existed at that period of life. I am inclined to believe that such was the fact.

Having spoken of the symptoms of this form of mental disease, the Society will naturally expect that I should enter into some details in regard to its treatment. I think, if the impairment be discerned in its early period, and prompt and energetic measures are adopted, the disease may, in the majority of cases, be cured. The great, the primary remedy, is REST FOR THE BRAIN, and as a sequence, rest for the MIND. Unless this condition be imperatively complied with, nothing—literally nothing—can be done. The medical treatment consists in a judicious adoption of those means which are calculated to restore a fibrinous or plastic state of the blood, and to promote its healthy circulation in the brain. The patient should live generously, and the digestive functions should be improved by the exhibition of gentle alteratives, tonics, and stimulants. After ramollissement has taken place, these remedies act by determining the blood to the portion of the brain where a defective supply exists, and, as it has been suggested, may act beneficially by causing the production of fibrine and cell-germs from the albumen of the blood. In non-inflammatory softenings, *rubefacients* must be applied, and the shower-bath used. The tonics found most beneficial have been *Zinc*, *Iron*, *Quinine*, and the vegetable acids. The patient will derive an advantage from the use of chalybeate aperient mineral waters. Small doses of phosphorus, perseveringly exhibited, have been found advantageous. I have, in some cases, given the nitrate of silver with benefit. A careful avoidance of mental excitement, and removing the individual from the scene of business, should be strictly enjoined, the patient carefully abstaining from all excesses, paying particular attention to dietetic rules, refraining from the use of wine, spirits, coffee, and spices. My principal object in bringing this subject before the profession, is not for the purpose of developing any novel views in reference to its pathology or treatment, but to point out the necessity of a careful and judicious exercise of those faculties with which God has endowed us. Length of life and sanity of mind are incompatible with *an excessive and continuous exercise and taxation of the functions of the brain.* There is, perhaps, no class of men more reckless of this physiological fact than the profession I have the honour to address. It is, indeed, lamentable to witness the devastations which have followed a non-recognition of this important law among some of the brightest ornaments of our profession. Let us be wise in time. As we rest the stomach when it presents evidence of its powers having been unduly strained, so let us allow the brain to repose when we feel conscious that its peculiar functions have been severely exercised, and the mind presents deviations from a normal state.

Savill & Edwards, Printers, Chandos Street, Covent Garden.

